# Low extracellular pH protects cancer cells from ammonia toxicity

**DOI:** 10.1038/s41420-025-02440-w

**Published:** 2025-04-03

**Authors:** Maria Dravecka, Ingvild Mikkola, Terje Johansen, Ole Morten Seternes, Jakob Mejlvang

**Affiliations:** 1 Cell Signalling and Targeted therapy, Department of Pharmacy, UiT The Arctic University of Norway, Tromsø, Norway; 2 Autophagy Research Group, Department of Medical Biology, UiT The Arctic University of Norway, Tromsø, Norway

**Keywords:** Cancer microenvironment, Cell death, Autophagy, Cell growth

## Abstract

Ammonia is a natural waste product of cellular metabolism which, through its lysosomotropic ability, can have detrimental effects on various cellular functions. Increased levels of ammonia were recently detected in the interstitial fluid of various tumours, substantiating that high ammonia concentrations are a pathophysiological condition in the tumour microenvironment, alongside hypoxia and acidosis. Since little is known about how cancer cells respond to elevated levels of ammonia in the tumour microenvironment, we investigated how a panel of cancer cell lines derived from solid tumours behaved when exposed to increasing concentrations of ammonia. We found that ammonia represses cell growth, induces genome instability, and inhibits lysosome-mediated proteolysis in a dose-dependent manner. Unexpectedly, we also found that small fluctuations in the pH of the extracellular environment, had a significant impact on the cytotoxic effects of ammonia. In summary, our data show that the balance of pH and ammonia within the interstitial fluids of cancerous tumours significantly impacts the behaviour and fate of cells residing in the tumour microenvironment.

## Introduction

The development of targeted cancer therapies necessitates the identification of molecular targets which can be based on any specific feature of the cancer that is uncommon in normal cells or tissue. One such feature is represented by the pathophysiological conditions arising in the tumour microenvironment due to the combined effect of cancer-associated metabolic reprogramming and decreased perfusion caused by improper tissue architecture. The best-characterised pathophysiological condition arising in the tumour microenvironment is reduced oxygen availability, known as hypoxia [1]. Hypoxia induces the expression of glucose transporters, glycolytic enzymes, and inhibitory kinases of the pyruvate dehydrogenase complex, which all serve to increase glycolysis in the cancer cells [2, 3]. The increased secretion of lactic acid derived from glycolysis, together with increased concentrations of CO_2_ produced from oxidative phosphorylation, causes acidification of the interstitial fluid which is referred to as tumour acidosis [4]. Since both acidosis and hypoxia have a wide impact on the proliferative and metastatic potential of cancer cells [4, 5], new therapeutic strategies are being developed to target these two pathophysiological conditions in the tumour microenvironment [6–8].

Besides anaerobic glycolysis, metabolic reprogramming of cancer cells also involves increased dependence on glutamine. Glutamine can serve both as an energy supply and a carbon/nitrogen source for the synthesis of nonessential amino acids, nucleotides and fatty acids [2]. During hypoxia, cancer cells preferentially use glutamine to provide carbon for fatty acid biosynthesis through reductive carboxylation to meet the demand for lipid synthesis [9, 10]. The increased flux of glutamine into α-ketoglutarate drives a constant release of ammonia. Although breast cancer cells in vitro have been shown to utilize some of this ammonia as a nitrogen source [11], a positive net production of ammonia is released into the interstitial fluids, causing ammonia concentrations in the tumour microenvironment to reach 1–10 mM [11–13]. Because these concentrations have been shown to reduce viability in various cell lines in vitro [14–18], ammonia is emerging as a novel pathophysiological condition of the tumour microenvironment. Yet, there is a paucity of data in the literature addressing how cancer cells are affected by extracellular ammonia.

Ammonia refers to the combined pool of mildly acidic ammonium ions (NH_4_^+^) and weakly basic ammonia gas molecules (NH_3_) which exists in an equilibrium largely governed by pH and temperature. Because NH_4_^+^ is a charged ion, it requires membrane-embedded transporter proteins to cross cellular membranes [19]. In contrast, NH_3_ can freely dissociate over membranes. The different biochemical properties of NH_4_^+^ and NH_3_, cause ammonia to have a lysosomotropic effect, which means it accumulates and alkalises acidic organelles [20]. The cytotoxic effect of ammonia is therefore likely related to its impact on the endolysosomal system which consists of various interconverting acidic vesicles with pH ranging from 6.7 to 4.7, i.e., early and late endosomes, endolysosomes, autolysosomes, phagolysosomes, autophagolysosomes and primary lysosomes [21, 22]. For example, the most abundant lysosomal proteases, cathepsins, are only activated when pH reaches a certain lower threshold [23, 24]. Consistently, degradation of substrates derived from the endocytic, autophagic, and phagocytic pathways can be reduced when cells are exposed to high levels (≥10 mM) of ammonia [16, 20]. In contrast, intermediate levels (1–4 mM) have been suggested to induce autophagy [12, 25–27], a process leading to lysosomal degradation of cytoplasm.

Recognising that ammonia levels in the tumour microenvironment may play a significant role in cancer progression, we investigated how different concentrations of ammonia affect various cancer cell lines in vitro. We found that ammonia inhibits lysosomal proteolysis in a dose-dependent manner, leading to an accumulation of proteins involved in endocytic, autophagic, and phagocytic pathways. This inhibition results in decreased cell growth, increased cell death and genome instability. Moreover, low extracellular pH, which is often present in the tumour microenvironment, counteracts the effects of ammonia. Therefore, the balance of pH and ammonia within the interstitial fluids of cancerous tumours can therefore have a significant impact on cancer progression and may potentially be targeted in future therapies.

## Results

### Ammonia inhibits cell growth at tumour-relevant concentrations

**Fig. 1:**
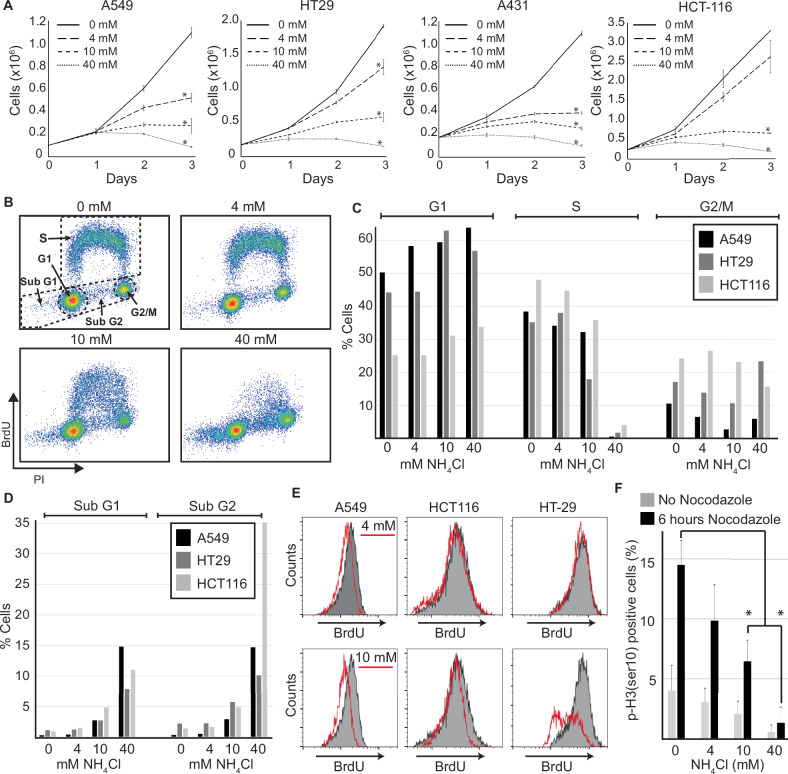
Extracellular ammonia reduces the growth of cancer cells in a dose-dependent manner. **A** Growth curves for indicated cell lines cultured in media with indicated concentrations of NH_4_Cl. Error bars display the standard deviation of biological triplicates. *(*p* < 0.01, two-tailed Student’s *t* test). Source data are provided in the Source Data file. **B**–**E** Indicated cell lines were cultured in media with indicated concentrations of NH_4_Cl for three days. Then, cells were subjected to 10 min of BrdU (20 μM) pulse labelling. Cells were subsequently analysed for BrdU and DNA content (PI, propidium iodide) by flow cytometry. The experiment was performed three times, yielding similar results. **B** Dot plot showing DNA content (PI), and BrdU incorporation (BrdU) for HT-29 cells. **C**, **D** Column chart showing the distribution of cells in each subpopulation defined in (**B**). **E** Histograms depicting BrdU incorporation in cells replicating DNA (S-phase cells). Grey histograms represents cells that have been cultured without NH_4_Cl. **F** A549 cells were grown in the presence of the depicted concentrations of NH_4_Cl for three days. Then, cells positive for Histone H3 phosphorylated at ser10 (p-H3(ser10)) were registered by immunofluorescence. Where indicated, cells were treated with 100 ng/ml Nocodazole to arrest cells in M-phase. Error bars represent the standard error of the mean. *(*p* < 0.05, as determined by two-tailed Student’s *t* test). Source data are provided in the Source Data file.

To investigate how ammonia affected growth of cancer cells, we cultured a panel of human carcinoma cell lines (A549, A431, HT29, and HCT116) in the presence of 0, 4, 10, and 40 mM of NH_4_Cl. The particular cell lines were chosen because they collectively represent a diverse range of cancer types with high prevalence. In addition, these cell lines are well-established and widely used in cancer research, allowing for better comparison and validation of our findings against existing literature. To minimize the artificial influx of ammonia from cell metabolism and spontaneous glutamine hydrolysis, we changed the media each day and used media containing GlutaMAX instead of L-glutamine. 4 mM NH_4_Cl moderately repressed growth in all cell lines except HCT116 (Fig. 1A). 10 mM NH_4_Cl strongly repressed growth in all cell lines, while cells exposed to 40 mM NH_4_Cl ceased to grow after 24 h and even caused a small decline in cell numbers over the following two days. To elucidate how ammonia suppresses cell growth, we analysed cell cycle distribution and the extent of DNA synthesis after cells had been cultured for three days in the presence of increasing concentrations of NH_4_Cl. Exposure to 4 mM NH_4_Cl did not have any noticeable effect on cell cycle distribution in any of the cell lines (Fig. 1B–D). 10 mM NH_4_Cl caused a small increase in cells located in the G1 phase and a decrease in cells undergoing replication (in S and G2/M phase). 10 mM NH_4_Cl also caused an increase in the number of cells in sub-G1 and an increase in cells that contained DNA between 2n and 4n but did not incorporate BrdU (sub-G2, Fig. 1B, D). At 40 mM NH_4_Cl, less than 5% of the cells incorporated BrdU and more than 20% of the cells were localized to sub-G1 and sub-G2. Interestingly, cells synthesised DNA slower when cultured in presence of NH_4_Cl (Fig. 1E). In A549 cells this was evident already at 4 mM, while all investigated cell lines displayed this feature at 10 mM. To get a more dynamic view of the cell cycle progression we measured how many cells entered M-phase during 6 h using phosphorylated histone H3 (ser10) as an M-phase marker (Fig. 1F). To prevent cells from advancing beyond M-phase we used nocodazole. This analysis revealed that NH_4_Cl exposure caused a dose-dependent decrease in cells reaching M-phase where only very few cells treated with 40 mM NH_4_Cl reached M-phase.

### Ammonia does not trigger the ATR/ATM-mediated checkpoint, nor does it cause apoptosis

**Fig. 2:**
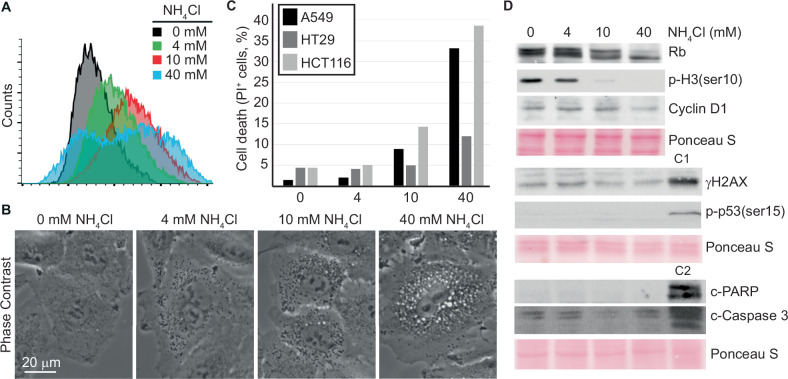
Reduced growth and cell death caused by extracellular ammonia is not coupled to ATR/ATM checkpoint signalling. **A** A549 cells were cultured for three days in the presence of indicated concentrations of NH_4_Cl. Cells were then analysed by flow cytometry. SSC; side scatter, *n* = 20.000). **B** A549 cells were cultured in media with the indicated concentrations of NH_4_Cl and then imaged in vivo by phase-contrast microscopy. **C** Cell death assay. After indicated cell lines had been cultured in media with indicated concentrations of NH_4_Cl for three days, cells were collected by trypsination, stained with propidium iodide, and analysed by flow cytometry (*n* = 20000). **D** A549 cells were cultured for three days in media with indicated concentrations of NH_4_Cl. Whole-cell lysates were prepared, and Western blot analysis was performed. Ponceau S was used as a loading and blotting control. C1 and C2 represent control lysates. C1; A549 cells cultured overnight in the presence of 50 μM Etoposide. C2; Jurkat cells subjected to heat shock (10 minutes at 44 °C followed by 6 hours at 37 °C). Molecular markers and densitometry analysis are shown in the Source Data file.

The flow cytometry analysis also revealed that the side scatter (SSC) signal increased in a dose-dependent manner in cells exposed to NH_4_Cl (Fig. 2A). Of note, SSC provides information about the internal complexity (i.e., granularity) of a cell. In agreement, when cells were observed under the microscope, NH_4_Cl caused a dose-dependent increase in the number of intracellular granules (Fig. 2B), indicating an accumulation of certain intracellular components. We furthermore noted that cell cultures treated with 10 and 40 mM produced more cellular debris, indicative of increased cell death (Supplementary Fig. S1). Measuring cell death by propidium iodide uptake, we found that cell death was induced by NH4Cl in a dose-dependent manner, leading to substantial cell death (10–40%) in all cell lines at 40 mM NH_4_Cl (Fig. 2C). The cell death was not associated with cleavage of PARP or Caspase 3 (Fig. 2D and Supplementary Fig. S2), indicating that NH_4_Cl induces necrosis rather than apoptosis. This conclusion was further supported by an annexin-based apoptosis assay (Supplementary Fig. S3). We next investigated whether NH_4_Cl exposure activated any DNA damage checkpoints by looking at the phosphorylation status of p53 and H2AX (phosphorylated form referred to as γH2AX). None of the tested concentrations of NH_4_Cl led to increased phosphorylation of p53 at Ser15 in any of the cell lines (Fig. 2D and Supplementary Fig. S2). However, in HT29 and HCT116 cells, 40 mM of NH_4_Cl led to an increase in γH2AX (Supplementary Fig. S2). Lastly, we checked whether ammonia exposure repressed proliferation through the G1/S cell cycle checkpoint mediated by the CDK4/6-Rb signalling hub. Whereas 4 and 10 mM NH_4_Cl did not affect the phosphorylation status of retinoblastoma protein (Rb), hypophosphorylated Rb was detected in cells cultured in the presence of 40 mM NH_4_Cl (Fig. 2D and Supplementary Fig. S2). This hypophosphorylation of Rb coincided with decreased expression of Cyclin D1, substantiating that lethally high concentrations of NH_4_Cl eventually cause cell cycle arrest in G1 through the CDK4/6-Rb signalling pathway. Taken together, this suggests that ammonia inhibits cancer cell growth in a dose-dependent manner, with IC50 values (based on three days of growth) ranging from 2 to 7 mM, and with an onset of cell death with concentrations exceeding 10 mM. Based on the diminished expression of phosphorylated H3 (at serine 10), the lack of ATR/ATM-mediated checkpoint signalling, and the increased prevalence of cells in sub-G2, we hypothesise that ammonia affects DNA replication in a way that causes mitotic catastrophe followed by necrosis. Thus, hyperammonemia in the tumour microenvironment likely contributes to genome instability in residing cancer cells.

### Ammonia causes increased expression of various components of the endolysosomal system

**Fig. 3:**
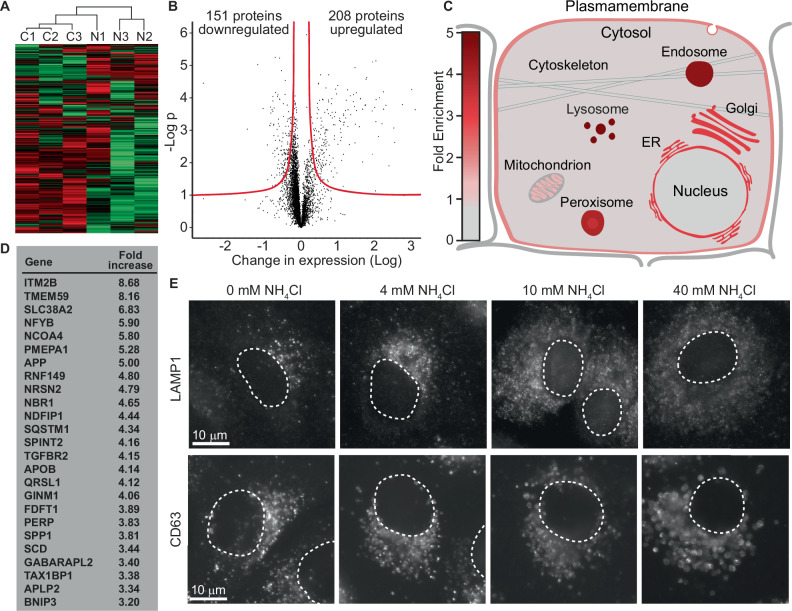
Extracellular ammonia causes accumulation of cellular components associated with the endolysosomal system. **A**–**D** Mass spectrometry-based quantitative proteomics was performed on A549 cells cultured for 24 h in the absence/presence of 4 mM NH_4_Cl. **A** Heatmap representation of differentially expressed proteins with hierarchical clustering of samples (control samples: C1-3, 4 mM NH_4_Cl samples: N1-3). Shades of red indicate up-regulated proteins; shades of green indicate down-regulated proteins. **B** Volcano plot of all identified proteins. The red line depicts the border of significance (see Material and Methods). **C** Depiction of fold enrichment of displayed cellular components based on gene ontology enrichment analysis. **D** Table listing the 25 most upregulated proteins. **E** A549 cells were cultured for two days in media with the indicated concentrations of NH_4_Cl. Cells were then fixed with MeOH, stained by immunohistochemistry, and analysed by fluorescence microscopy. Dotted lines depict nuclei.

Although ammonia has long been reported to cause a swelling effect on certain intracellular organelles, ammonia’s effect on the proteome has never been investigated, and it is unknown which specific proteins and cellular components are affected. We therefore decided to perform a mass spectrometry-based quantitative proteomic study to determine how 24-hour exposure to 4 mM NH_4_Cl affected the proteome in A549 cells. Based on three biological replicates, we identified the relative change in expression of 5 962 proteins (Fig. 3A, B and Supplementary Datasheet 1A). 208 proteins increased significantly in expression, while 151 proteins decreased (Supplementary Datasheet 1B, C). Gene ontology enrichment analysis (PANTHER Overrepresentation Test [28]) of all proteins with significantly increased expression, revealed that the cellular components, lysosome, endosome, peroxisome, endoplasmic reticulum, Golgi apparatus, and plasma membrane were significantly enriched (Fig. 3C and Supplementary Datasheet 1D). These data suggest that ammonia predominantly affects the endolysosomal system and that ammonia causes an accumulation of proteins associated with the endosomal system. In agreement, culturing cells in the presence of NH_4_Cl clearly affected lysosomal and endosomal vesicles (Fig. 3E). In cells that had not been exposed to NH_4_Cl, the lysosomal marker LAMP1 was localized in small distinct foci, predominantly in the Golgi region, but with increasing concentrations of NH_4_Cl, LAMP1 foci increased in numbers, and their localization expanded to cover the whole cytoplasm. CD63-positive vesicles (endosomes) increased in size with increasing concentrations of NH_4_Cl, and at 40 mM NH_4_Cl, CD63 localized in a very homogeneous population of vesicles with a diameter of approximately 2 μm. Performing gene ontology enrichment analysis on the list of proteins that increased more than 3-fold (Fig. 3D), identified the autolysosome as one of the most enriched cellular components (Supplementary Datasheet 1E). We next chose to investigate how ammonia affected this component in more detail.

### Ammonia inhibits lysosome-mediated degradation of the autophagic marker LC3B

**Fig. 4:**
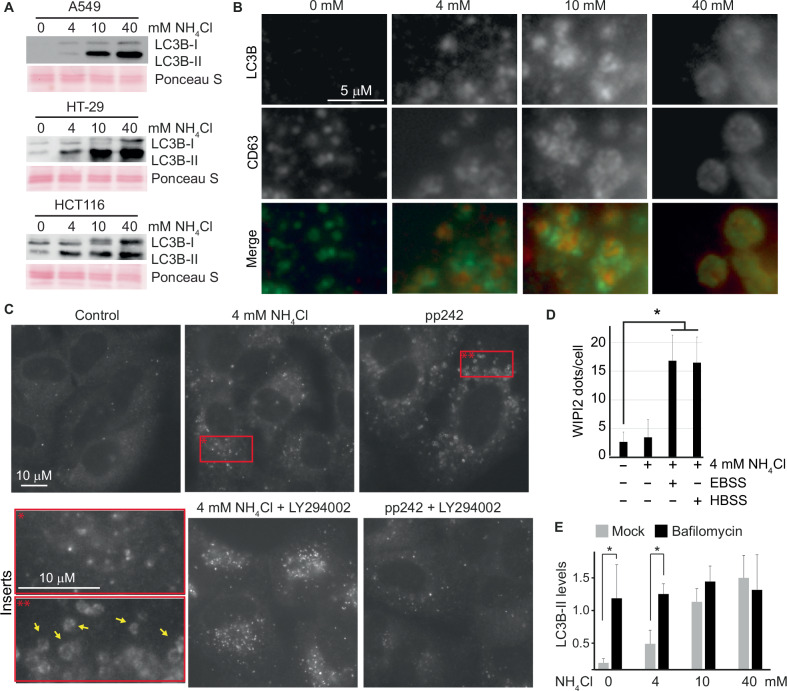
Extracellular ammonia does not induce autophagy but inhibits lysosome-mediated protein degradation of LC3B in a dose-dependent manner. **A** Depicted cell lines were cultured for three days in media with indicated concentrations of NH_4_Cl. Whole-cell lysates were prepared, and Western blot analysis was performed. Ponceau S was used as a loading and blotting control. Molecular markers and densitometry analysis are shown in the Source Data file. **B** A549 cells were cultured for 24 h in media with the indicated concentrations of NH_4_Cl. Then, they were fixed with MeOH, stained by immunohistochemistry, and analysed by fluorescence microscopy. In the merged panel, green represents CD63 while red represents LC3B. Less magnified images including the nucleus are shown in Supplementary Fig. S5. **C** A549 cells were cultured for 4 h in the indicated presence of 4 mM NH_4_Cl, 250 nM pp242, and 25 μM LY294002. Then, the cells were fixed with MeOH, stained by immunohistochemistry, and analysed by fluorescence microscopy. Red squares represent the area shown with increased magnification in the inserts. Yellow arrows highlight classical autophagosomes. **D** A549 cells were cultured for one day in media with 4 mM NH_4_Cl. Where indicated, cells were subjected to 4 h of starvation (HBSS or EBSS) in the continued presence of NH_4_Cl. Then, the cells were fixed with MeOH and stained with an antibody targeting WIPI2. The quantification of WIPI2 dots per cell are shown in the figure while representative images used for the quantification are shown in Supplementary Fig. S7. Error bars represent the standard deviation (*n* > 20). *(*p* < 0.01, two-tailed Student’s *t* test). Source data are provided in the Source Data file. **E** A549 cells were cultured for 5 h in media with the indicated concentrations of NH_4_Cl, either with or without bafilomycin (200 nM). The figure depicts relative LC3B-II levels based on quantitative densitometric analysis of Western blots. Error bars represent the standard error of 5 biological replicates. *(*p* < 0.05, as determined by two-sided Welch’s *t* test). Source data are provided in the Source Data file.

LC3B is a general marker for autophagic membranes [29, 30]. It is conjugated to phosphatidylethanolamine anchored in the autophagosomal membrane and serves as a recruitment platform for various cargo receptors which add selectivity to the autophagic process [31]. This latter function is not restricted to macroautophagy (formation and processing of autophagosomes) but is also operative in endosomal microautophagy [32]. LC3B, and other ATG8 family proteins, are also conjugated to single membranes in non-canonical autophagy processes that are triggered by a variety of stimuli [33]. LC3B strongly increased in expression with increasing exposure to NH_4_Cl (Fig. 4A), and the increase was detected already after 1–2 hours of exposure (Supplementary Figs. S4–5). In contrast to LAMP1 and CD63, LC3B was hardly detectable by immunofluorescence when cells were cultured in media without ammonia (Fig. 4B and Supplementary Fig. S5). However, by adding as little as 1 mM NH_4_Cl to the growth media, LC3B puncta appeared (Supplementary Fig. S7), and with increasing concentrations of NH_4_Cl, LC3B puncta increased in numbers (Fig. 4B). These LC3B puncta had a strong tendency to localize to areas positive for CD63, and analysed by high magnification, LC3B puncta seemingly localised within CD63-positive vesicles (Fig. 4B and Supplementary Fig. S6). The increase in LC3B did not reflect a stimulation of macroautophagy. Firstly, because the formation of LC3B puncta stimulated by NH_4_Cl was not inhibited by pharmacological inhibition of macroautophagy by LY294002 (Fig. 4C), and secondly, because NH_4_Cl did not cause any increase in WIPI2 puncta, an early marker for autophagosome formation [34] (Fig. 4D). As high extracellular concentrations (≥10 mM) of ammonia have previously been shown to inhibit lysosome-mediated proteolysis [20, 29], we next tested how different concentrations of ammonia inhibited the basal lysosome-mediated degradation of LC3B. To this end, we compared how much LC3B increased in expression when cells were exposed to NH_4_Cl, to how much it increased when lysosomal-mediated degradation was completely blocked by Bafilomycin A1. Exposing cells to ammonia alone, led to a dose-dependent increase in LC3B expression (Fig. 4E). Concentrations of both 10 and 40 mM NH_4_Cl resulted in an increase comparable to that caused by Bafilomycin A1, indicating that these concentrations blocked lysosome-mediated degradation. This demonstrates that the lysosome-mediated degradation of LC3B gradually decreases with increasing concentrations of NH_4_Cl until it ceases when extracellular NH_4_Cl reaches a threshold (approximately 10 mM).

### Low extracellular pH counteracts the lysosomotropic effect of extracellular ammonia

**Fig. 5:**
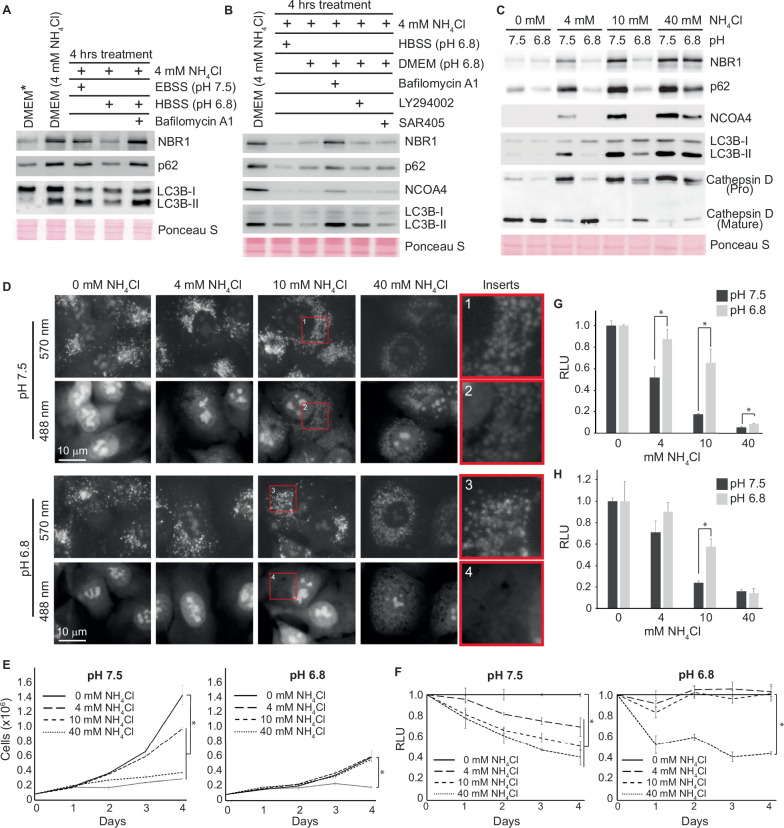
Low extracellular pH reduces the growth inhibitory effect of extracellular ammonia. **A**, **B** A549 cells were cultured for one day in media (DMEM) with 4 mM NH_4_Cl and then subjected to indicated treatments for 4 h. DMEM* represents cells cultured in DMEM without NH_4_Cl. Western blot analysis of indicated proteins. Bafilomycin A1, 200 nM. LY294002, 10 μM. SAR405, 1 μM. Molecular markers and densitometry analysis are shown in the Source Data file. **C** A549 cells were cultured for two days in media with the indicated pH and indicated concentration of NH_4_Cl. Western blot analysis of indicated proteins. Molecular markers and densitometry analysis are shown in the Source Data file. **D** A549 cells were cultured for two days in media with the indicated pH and indicated concentration of NH_4_Cl. Then, cells were stained with Acridine Orange for 5 min and subsequently analysed by fluorescence microscopy (in vivo). Red squares depict the regions that are magnified to the far right (inserts). **E** Growth curves based on cell counting. A549 cells were cultured in media with either pH 7.5 or 6.8 and with the indicated concentrations of NH_4_Cl. Error bars represent the standard deviation of 3 replicates. *(*p* < 0.05, two-tailed Student’s *t* test). **F** Cell viability assay (CellTiter-Glo). A549 cells were exposed to indicated concentrations of NH_4_Cl while being cultured in media with either pH 7.5 or 6.8. Error bars represent the standard deviation of 3 replicates. *(*p* < 0.05, two-tailed Student’s *t* test). Cell viability assay. HT29 (**G**) and A431 (**H**) cells were cultured in media with either pH 7.5 or 6.8 with the indicated concentrations of NH_4_Cl. After three days cell viability was measured by CellTiter-Glo. *(*p* < 0.05, two-tailed Student’s *t* test). **E**–**H** Source data are provided in the Source Data file.

In addition to LC3B, we also observed an accumulation of its associated cargo receptors p62, NBR1, TAXBP1, CALCOCO2 and the receptor for ferritinophagy, NCOA4 (Fig. 3D and Supplementary Datasheet 1B). Since we previously identified these proteins to be substrates for starvation-induced endosomal macroautophagy [32], we tested how intermediate concentrations of ammonia affected this process. Cells cultured for 24 h in the presence of 4 mM NH_4_Cl were starved for amino acids and serum (in the continued presence of 4 mM NH_4_Cl) using either Earle’s balanced salt solution (EBSS) or Hanks’ balanced salt solution (HBSS). Much to our surprise, starvation in EBSS had minimal effect on the expression of LC3B, NBR1, p62, and NCOA4 (hereafter referred to as autophagic substrates), while HBSS caused a substantial reduction (Fig. 5A). An inspection of the chemical composition of the two starvation buffers revealed that they mainly differed in the content of sodium bicarbonate (0.35 g/L in HBSS vs. 2.2 g/L in EBSS), which is instrumental for buffering pH. We therefore compared the pH of EBSS and HBSS after they had been acclimatized to 5% CO_2_ and 37 °C. This comparison disclosed that while EBSS had a pH of 7.5, HBSS had a much lower pH of 6.8. When we supplemented HBSS with sodium bicarbonate to reach 2.2 g/L, the pH increased to 7.5, and we no longer observed a degradation of the substrates. Moreover, lowering the pH of the media to 6.8 caused a similar stimulation of lysosome-mediated degradation of autophagic substrates compared to HBSS in cells exposed to 4 mM NH_4_Cl and this degradation was minimally affected by pharmacological inhibition of macroautophagy by LY294002 or SAR405 (Fig. 5B). We next tested whether the accumulation of autophagic substrates upon NH_4_Cl exposure was prevented when the pH of the full media was lowered to 6.8. In sharp contrast to cells cultured at pH 7.5, cells cultured at pH 6.8 did not accumulate autophagic substrates when exposed to up to 10 mM NH_4_Cl (Fig. 5C and Supplementary Fig. S9). However, at 40 mM, autophagic substrates accumulated almost to the same level as for cells cultured at pH 7.5. Western blot analysis of one of the central lysosomal proteases, Cathepsin D, revealed that the maturation of Cathepsin D was inhibited by NH_4_Cl in a dose-dependent manner when cells were cultured at pH 7.5 (Fig. 5C). The proteolytic maturation of Cathepsin D takes place inside endolysosomal vesicles when these reach approximately pH 5 [23, 24]. The impaired maturation of Cathepsin D could therefore reflect that NH_4_Cl exposure indirectly impairs Cathepsin D maturation by preventing proper acidification of endolysosomal vesicles. To test this hypothesis, we stained living cells cultured at pH 7.5 with the fluorescent dye Acridine Orange. As a monomer, Acridine Orange emits green light (488 nm), but when it is protonated in an acidic environment, Acridine Orange forms aggregates that emit red fluorescence (570 nm) [35]. In the absence of NH_4_Cl, red light was emitted from small puncta in the cytosol, and green light was emitted from nuclei/nucleoli due to its affinity for DNA and RNA (Fig. 5D). With the increasing presence of NH_4_Cl, the red puncta in the cytoplasm became both larger and dimmer and started to emit green light (see inserts 1 and 2 in Fig. 5D). In contrast, when cells were cultured in media with pH 6.8, concentrations up to 10 mM NH_4_Cl barely had any effect on the Acridine Orange staining pattern. Similarly, lowering the pH of the media reduced the impact of NH_4_Cl on Cathepsin D maturation (Fig. 5C).

### Low extracellular pH counteracts the effects of extracellular ammonia

Lastly, we questioned whether the growth inhibitory effects of NH_4_Cl were also lessened when cells were cultured in media with pH 6.8. As previously reported [36], lowering the pH of the growth media caused a small decrease in growth (from a 2.06-fold increase per day (±0.18) to a 1.65-fold increase per day (±0.21)). In striking contrast to A549 cells cultured at pH 7.5, 4–10 mM NH_4_Cl did not supress growth of A549 cells cultured at pH 6.8 (Fig. 5E, F).

**Fig. 6:**
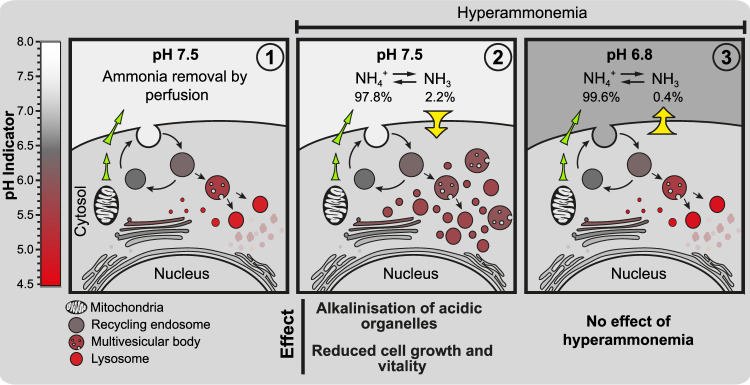
Model summarizing the effect of hyperammonemia at normal physiological pH (pH 7.5) versus low extracellular pH (pH 6.8). Panel 1. Increased glutaminolysis leads to a positive net production of NH_4_^+^, which is exported from the mitochondria to the extracellular space (green arrows). When perfusion of the interstitial fluid is normal, ammonia is constantly removed from the microenvironment. Panel 2. When perfusion is abrogated, ammonia accumulates in the extracellular space. Under normal physiological pH, 2.2% of the total ammonia pool will be in the form of NH_3_. As an alkaline gas, NH_3_ can traverse the plasma membrane and enter the more acidic cytosol (yellow arrow). When the influx of NH_3_ exceeds the efflux of NH_4_^+^, ammonia will accumulate in the cytosol, where it leads to a dose-dependent alkalinization of acidic organelles, which impedes their turnover and function. This indirectly represses cell growth and vitality. Panel 3. When the extracellular pH is 6.8, less (0.4%) of the total ammonia pool will be in the form of NH_3_. Therefore, less NH_3_ will be available to enter the cytoplasm. In addition, low extracellular pH instigates a reversed pH gradient, impeding the flux of NH_3_ to the cytosol (yellow arrow). Consequently, high extracellular ammonia levels are largely effectless when cells are subjected to low extracellular pH.

However, when the concentration increased to 40 mM, cell growth was inhibited to the same degree in media with pH 6.8 compared to media with pH 7.5. Similarly, lowering the pH of the growth media significantly improved the growth of A431 and HT-29 cells exposed to up to 10 mM NH_4_Cl (Fig. 5G, H). In summary our data corroborate that at pH 7.5, extracellular ammonia in the form of NH_3_ diffuses down a pH gradient into the more acidic cytosol and then into the even more acidic intracellular compartments where it becomes protonated (for a more elaborate explanation, see Fig. 6). This ion-trapping effect leads to alkalization of the acidic intracellular organelles such as endolysosomal vesicles. In a dose-dependent manner, the alkalization impairs the maturation and function of endolysosomal proteases, thereby affecting the turnover of endolysosomal vesicles, which eventually leads to an accumulation of semi-acidified vesicles. The vesicles increase in size (swell) due to osmotic pressure caused by an accompanying increase in chloride ions [37]. In combination, this affects the many various processes mediated by the endolysosomal system, including energy and amino acid sensing, signal transduction, ionic homeostasis, mitosis, and lysosome exocytosis [38, 39], in addition to protein synthesis [20, 40], consequently resulting in reduced growth and vitality (Fig. 6). Interestingly, our results show that the cytotoxic effect of ammonia is inhibited when the extracellular pH is lowered to pH 6.8. We envision that this effect is due to two specific reasons. Firstly, because a lower extracellular pH will shift the NH_4_^+^/NH_3_ equilibrium in favour of NH_4_^+^, leading to a decreased concentration of extracellular NH_3_. For example, 2.2% of ammonia will be in the form of NH_3_ at pH 7.5, in contrast to 0.4% at pH 6.8 (based on the Henderson–Hasselbalch equation). Secondly, an extracellular pH lower than the intracellular pH results in a reversed pH gradient between the two compartments, thus reducing the ion-trapping effect [8]. The model of how ammonia increases the pH of acidic intracellular compartments is far from novel [15], but the finding that a relatively small decrease in extracellular pH has a tremendous effect on ammonia’s cytotoxicity is new.

The impact of increased exposure to NH_4_Cl may not only be caused by its lysosomotropic effect but could also be due to ammonia’s metabolic properties, as it is involved in numerous biochemical reactions. However, since the effects of NH_4_Cl to a large extent are phenocopied by the lysosomotropic drug chloroquine [41–43], whose pKa is similar to NH_4_^+^, we believe that the effect of NH_4_Cl is mainly due to its lysosomotropic effect. Consistently, the cytotoxic effect of chloroquine was recently shown to be diminished when the extracellular pH was lowered to 6.8 [42, 44].

## Discussion

Our results show that physiologically relevant levels of ammonia can inhibit the growth of cancer cells. However, it was recently shown that extracellular ammonia can also inhibit T cells [13], and our own unpublished data show that extracellular ammonia affects untransformed fibroblasts (BJ-1 cells) similarly to the panel of cancer cell lines we investigated. It is therefore a complex question whether ammonia’s cytotoxicity can be successfully exploited to treat solid cancer tumours. On one hand, the cytotoxicity of ammonia inhibits the growth of cancer cells; on the other hand, it suppresses the immune response attempting to fight it.

Currently, increasing the pH of the tumour microenvironment is thought to present a multi-faceted approach to enhance cancer treatments, as an acidic tumour microenvironment can stimulate carcinogenesis as well as decrease the efficacy of the immune system and the uptake of certain chemotherapeutic agents [8]. Our finding that the cytotoxicity of ammonia is strongly affected by pH should therefore be considered in this regard, as it will naturally affect the outcome of treatments based on regulating the pH of the tumour microenvironment. Given that the accumulation of ammonia within the tumour microenvironment is directly caused by the elevated metabolic degradation of glutamine inside the cancer cells, our findings will naturally also have to be considered with respect to chemotherapies based on limiting cancer cells’ utilization of glutamine. As glycolysis causes the accumulation of lactic acid and glutaminolysis causes the accumulation of ammonia, our finding apparently reveals an until-now unappreciated interrelation between glycolysis and glutaminolysis, where elevated glycolysis is a prerequisite for elevated glutamine metabolism in an environment with impaired perfusion.

Our main finding, that ammonia toxicity is highly dependent on extracellular pH, highlights an important caveat in attempts to elucidate the effects of different pathophysiological conditions in the tumour microenvironment through studies focusing on a single, isolated pathophysiological condition. For example, the effect of hypoxia is widely studied in vitro by culturing cells in the diminished presence of O_2_ (1%), but with normal levels of CO_2_ (5%), and no addition of lactate [45]. However, hypoxia in the tumour microenvironment occurs in the presence of hypercapnia (elevated CO_2_) and elevated levels of lactic acidosis [6, 46], and several studies have actually shown that the hypoxia-induced cellular response is highly influenced by lactic acidosis [47–49]. Similarly, we find that the cytotoxic effect of ammonia is dependent on the extracellular pH. Since acidosis and hyperammonemia likely are spatially linked, we can only conclude that a much more refined understanding of the spatial balance between ammonia and pH is required to predict to what degree ammonia exerts cytotoxicity in the tumour microenvironment. The current estimates of ammonia concentrations in the interstitial fluid of the tumour are limited to average measurements [11–13]. Naturally, this implies that concentrations could be much higher in the areas of the tumour with the lowest perfusion. Several studies have shown that LC3B accumulates in the more hypoxic/acidic regions of the tumour [42, 50, 51], furthest away from functional capillaries where cancer cell growth is slowest. Indeed, this observation could reflect an increase in hypoxia-induced autophagy [51], but they could equally well suggest that hyperammonemia and acidosis in these areas reach a balance where ammonia inhibits lysosome efficacy and growth of the residing cells.

While the cell lines generally exhibited similar reactions to ammonia exposure, there were some variations in both susceptibility and response. As we have shown, even minor changes in both pH and ammonia concentrations can significantly impact cell behaviour. Differences in the endogenous release of ammonia through glutaminolysis and variations in intracellular pH among cell lines are therefore likely a major contributor to the observed differences in susceptibility. In A549 cells, necrosis induced by high extracellular ammonia levels was not associated with increased expression of γH2AX, consistent with the absence of ATR/ATM-mediated DNA damage signalling. In contrast, γH2AX levels increased in both HCT116 and HT29 cells when exposed to high ammonia concentrations. Our current hypothesis suggests that the increase in γH2AX in HCT116 and HT29 cells may be facilitated by DNA-PK during the late stages of necrosis, independent of the ATR/ATM pathway [52]. However, the discrepancy in γH2AX response between cell lines remains unexplained, as A549 cells are known to possess fully functional p53, ATR, ATM, and DNA-PK [53]. We found no evidence suggesting that high ammonia levels induce apoptosis, leading us to conclude that the observed cell death occurs as necrosis. Yet, our investigations are currently too limited to provide detailed insights into the mechanisms and types of necrosis triggered.

In line with lysosomes being the catabolic end stations of endocytic, autophagic and phagocytic pathways, we found that ammonia in a dose-dependent manner caused an accumulation of component involved in these pathways. For example, the expression of transmembrane receptor amyloid precursor protein (APP), which is endocytosed and subsequently processed in the lysosomes [54], increased 5-fold in cells exposed to 4 mM NH_4_Cl (see Supplementary Datasheet 1B). Consistently, Komatsu and colleagues also found that ammonia represses the degradation of APP [55]. However, we predominantly focused on elucidating how ammonia affected autophagy and found that the lysosomal-mediated protein degradation of LC3B was blocked by 10–40 mM NH_4_Cl in all tested cell lines. Furthermore, intermediate concentrations (1–4 mM), which only mildly affected cell proliferation, inhibited the lysosomal mediated degradation of LC3B in a dose-dependent manner. Several reports [12, 25–27] have suggested that intermediate concentrations of ammonia can induce autophagy. Common to these reports is that they predominantly base their conclusions on autophagic flux measurements estimated by the GFP-LC3 cleavage assay. As described in the “Guidelines for the use and interpretation of assays for monitoring autophagy (4th edition)” [29] ^(see Figure 12 therein)^, the GFP-LC3 cleavage assay can easily be misinterpreted, and in mammalian cells, the assay has actually been reported to detect impairment of lysosomal efficacy caused by increased lysosomal pH [56]. In this light, we find that the experimental results of the aforementioned reports, also reflect that ammonia, in a dose-dependent manner, increases lysosomal pH and thereby represses lysosomal proteolysis. We found no evidence suggesting that ammonia increases the flux of LC3B, nor did we find any evidence suggesting that basal macroautophagy contributed to the accumulation of LC3B in our model system. We therefore suspect that the accumulation of autophagic substrates taking place when cells are exposed to intermediate concentrations of extracellular ammonia, is caused by a dose-dependent inhibition of a basal autophagic process other than macroautophagy. Since the investigated substrates have previously been identified as substrates of endosomal microautophagy [32] we foresee that the basal autophagic activity repressed by ammonia foremostly represents basal endosomal microautophagy.

The maintenance of lysosomal pH depends on a dynamic equilibrium of H^+^ influx and efflux as well as counter-ion movement across the lysosomal membrane [57]. The influx is achieved by the V-ATPase, while the efflux is mediated by leakage through the lysosomal cation channel TMEM175 [58]. Counter-ion movement is necessary to dissipate the membrane potential arising from the V-ATPase activity, and is mediated by multiple ion channels and transporters in the lysosomal membrane, which allow the influx and efflux of the counter-ions Cl^-^, K^+^ and Na^+^ [57]. Growing evidence suggests that lysosomal pH is not simply maintained in a strict steady state but can be adjusted to fine-tune lysosomal function. For example, amino acid starvation enhances the assembly of the V-ATPase [59, 60], and lysosome-associated membrane protein 1 and 2, regulates the leakiness of TMEM175 [61]. Similarly, we envision that intracellular ammonia could be instrumental in regulating lysosome function. This is fully consistent with the recent finding that glutaminolysis, through its metabolic release of intracellular ammonia, represses lysosomal degradation by increasing the lysosomal pH [37, 62]. Furthermore, SLC12A9 was recently found to facilitate NH_4_^+^/Cl^-^ co-transport from the lysosome lumen to the cytosol [37]. In SLC12A9 knockout cells, both Cl^-^ and metabolically derived NH_4_^+^ accumulated in enlarged, semi-acidified lysosomal vesicles. This demonstrates that ammonia not only affects the lysosomal pH but also causes an osmotic imbalance in endolysosomal vesicles, which directly affects lysosomal trafficking, fusion and fission events [57]. A potentially lucrative therapeutic strategy could therefore be presented by pharmacologically inhibiting the efflux of ammonia from the cytosol, potentially by targeting SLC12A9. In theory, this would be specifically harmful to cells with elevated utilization of glutaminolysis, such as cancer cells.

## Materials and methods

### Cell culture and chemical treatments

A549 cells were cultured in DMEM with 1000 mg/L glucose (SIGMA#D5546) supplemented with serum (9%), penicillin (90 U/ml), streptomycin (10 μg/ml), and GlutaMAX (Gibco, 0.9X). In experiments where pH was adjusted to 7.5, A549 cells were cultured in DMEM (SIGMA#D5030) supplemented with serum (9%), penicillin (90 U/ml), streptomycin (10 μg/ml), GlutaMAX (Gibco, 0.9X), glucose (0.9 mg/ml) and sodium bicarbonate (1.29 g/L). In experiments where pH was adjusted to 6.8, A549 cells were cultured in DMEM (SIGMA#D5030) supplemented with serum (9%), penicillin (90 U/ml), streptomycin (10 μg/ml), GlutaMAX (Gibco, 0.9X), glucose (0.9 mg/ml) and sodium bicarbonate (0.3 g/L). In experiments where pH was adjusted to 7.5, A431 cells were cultured in DMEM (SIGMA#D5030) supplemented with serum (9%), penicillin (90 U/ml), streptomycin (10 μg/ml), GlutaMAX (Gibco, 0.9X), glucose (3.9 mg/ml), sodium pyruvate (0.9 mM), and sodium bicarbonate (1.29 g/L). In experiments where pH was adjusted to 6.8, A549 cells were cultured in DMEM (SIGMA#D5030) supplemented with Serum (9%), penicillin (90 U/ml), streptomycin (10 mg/ml), GlutaMAX (Gibco, 0.9X), glucose (3.9 mg/ml), sodium pyruvate (0.9 mM) and sodium bicarbonate (0.3 g/L). In experiments where pH was adjusted to 7.5, HT-29 and HCT-116 cells were cultured in McCoy′s 5 A Medium (SIGMA#M4892) supplemented with serum (9%), penicillin (90 U/ml), streptomycin (10 mg/ml) and sodium bicarbonate (1.34 g/L). In experiments where pH was adjusted to 6.8, HT-29 and HCT-116 cells were cultured in McCoy′s 5 A Medium (SIGMA#M4892) supplemented with Serum (9%), penicillin (90 U/ml), streptomycin (10 μg/ml), and sodium bicarbonate (0.38 g/L). All cell lines were cultured in HERACELL CO_2_-incubator in the presence of 5% CO_2_, and at 37 °C. During experiments, the media was changed each day. Before each media change, the media had been acclimatized in the CO_2_-incubator for a minimum of 1 h to reach the desired pH and temperature. Similarly, were Hanks’ Balanced Salt Solution (HBSS, SIGMA#H8264) and Earle′s Balanced Salts Solution (EBSS, SIGMA#E2888) were acclimatized in the CO_2_-incubator before use. All cell lines were regularly tested for mycoplasma contamination. All cell lines are derived from the American Type Culture Collection (ATCC). Where indicated, cells were treated with 200 nM bafilomycin A1 (Baf), 10 μM LY294002, 250 nM pp242, 1 μM SAR405, 1 μM wortmannin, 1 μM PIK-III, 100 ng/ml nocodazole, 100 nM LysoTracker^TM^ Red DND-99 (Molecular Probes), and 1 μg/ml acridine orange.

### Detection of apoptotic and dead (PI+) cells

For apoptosis detection, we used an APC Annexin V Apoptosis Detection Kit with PI (Biolegend). Cells were harvested by trypsinisation, washed twice with PBS (+1%BSA), and resuspended in 200 μl Annexin V Binding Buffer. Then, 5 μl of APC annexin V and 10 μl of Propidium Iodide (PI) solution were added, followed by an incubation for 15 min in the dark. Subsequently, 400 μl of Annexin V Binding Buffer was added before samples were analysed by flow cytometry on a BD LSRFortessa^TM^ Cell Analyzer. Data were processed using FlowJo^TM^ 10 software.

### Viability with CellTiter-Glo

Cells were seeded into 96-well plates and cultured as indicated. Following treatments, the CellTiter-Glo Luminescent Cell Viability Assay (Promega) was used to measure ATP levels, as instructed by the manufacturer.

### Western blot analysis

Samples for Western blotting were harvested in 1X SDS buffer (50 mM Tris, pH 6.8, 2% SDS, and 10% glycerol) and boiled for 5 min. Then protein concentrations were measured using BCA Protein Assay Kit (23227; Pierce) and lysates were calibrated. Bromophenol blue and DTT were added to final concentrations of 0.1% and 100 mM, respectively, before samples were boiled and run on SDS-PAGE gels at 120/160 V for 90 min. For blotting, a semidry transfer was used at 100 mA per blot for 1 hour using a buffer containing 14 mM glycine, 48 mM Tris, 0.03% SDS, and 0.15% ethanol. Membranes were stained with Ponceau S before blocking in 5% dry milk in 1X PBS-T for 30 min. Incubation with the primary antibody was performed overnight at 4 °C. Membranes were washed six times with 1X PBS-T before incubating with the secondary antibody for 1 hour at room temperature. Membranes were washed six times using 1X PBS-T. Membranes were developed using SuperSignal West Femto Chemiluminescent Substrate (34095; Pierce) and SuperSignal West Pico PLUS Chemiluminescent Substrate (34078; Pierce) on Hyperfilm ECL (18 ×24; GE28-9068-37; GE Healthcare) in a Curix 60 (AGFA) or by chemiluminescence detection using the ImageQuant LAS 3000 (GE Healthcare).

### Antibodies

The following primary antibodies were used in these studies: BrdU (BD Pharmingen, B44), LC3B (Sigma; L7543), NBR1 (Santa Cruz, sc-130380), NCOA4 (Sigma, SAB-1404569), p62/SQSTM1 (BD Biosciences, #610833), PARP (Cell Signalling, #9542), Cathepsin D (Abcam, ab75852), Rb (BD Pharmingen, #14001A), p-p53(Ser15) (Abcam, ab5176), p-H3(Ser10), gH2AX (Cell signalling, #2577, Cyclin D (Santa Cruz, Sc-753), cleaved Caspase 3 (Cell Signalling, #9661), CD63 (DSHB, H5C6), LAMP1 (DSHB, H4A3), and WIPI2 (Abcam, ab105459). The following secondary antibodies were used; HRP-conjugated goat anti-rabbit and anti-mouse (BD Pharmingen #554021 and #554002). Alexa Fluor 568- and 488-conjugated antibodies were purchased from Life Technologies and used at a 1:1000 dilution.

### Cell cycle analysis with propidium iodide and BrdU

For BrdU/PI labelling, cells were pulse-labelled for 10 min with 10 μM BrdU (Sigma#5002). Following trypsinization, cells were fixed in ice-cold 70% ethanol for 1 h and then washed once with PBS (+1% serum). Cell pellets were treated with 2 M HCl for 20 min at room temperature before three volumes of 0.1 M sodium borate (pH 8.5) were added for 2 min. Then, cells were washed twice with PBS (+1% serum) and incubated with BrdU antibody (1:20, BD Biosciences cat# 347580) for 1 h. Following two washes with PBS (+1% serum), cell pellets were incubated for 1 h with Alexa Fluor 488-conjugated anti-mouse in PBS (+1% serum). After two more washes with PBS (+1% serum), cell pellets were incubated with propidium iodide (50 μg/ml) and RNase (0.25 mg/ml) in PBS for 45 min. All incubations were done in the dark. Samples were finally analysed by flow cytometry on a BD LSRFortessa^TM^ Cell Analyzer, and data were processed using FlowJoTM 10 software.

### Immunocytochemistry and microscopy

Cells grown on round 12 mm coverslips were fixed for 10 min in cold (−20 °C) methanol and rinsed twice with cold PBS. They were then incubated in 2% BSA in PBS-T for 15 min at room temperature to block nonspecific binding. Subsequent incubations with the indicated primary antibodies and fluorescence-conjugated secondary antibodies were performed in PBS-T (containing 1% BSA) at room temperature for 1 h. Coverslips were rinsed four times for 2 min each with PBS-T following both incubations. Coverslips were subsequently stained with DAPI (0.25 ng/ml) and mounted using VECTASHIELD mounting medium.

For microscopy, we used a DeltaVision Elite microscope (GE) running SoftWoRx 1.0 software utilizing either a 60× or 100× objective. Image channels were acquired sequentially using appropriate filter sets for DAPI, Alexa Fluor 568, and 488. Ordinary phase-contrast micrographs were obtained using an AX10 microscope (Zeiss) equipped with a Retiga 6000 monochrome camera. In all experiments, images shown in individual panels were acquired using identical exposure times or scan settings and adjusted identically for brightness and contrast using Photoshop CS5 (Adobe).

### Proteomic analysis by mass spectrometry

A549 cells were cultured in the absence/presence of 4 mM NH_4_Cl for 24 h. Then, 2 ×10^6^ cells were lysed in 100 µl of lysis solution (1 M Urea, 0.5% SDC, and 100 mM TEAB) and sonicated for 25 cycles (1 min on, 30 s off) with 100% amplitude in a cup horn sonicator with a water cooler (cup horn/watercooler: Qsonica. Sonicator: Fisherbrand FB705 sonicator, Fisher). Aliquots of 70 µg protein were taken out, and proteins were reduced by DTT (5 mM) followed by alkylation with iodoaceamide (15 mM). To remove excess IAA, a DTT solution corresponding to a final concentration of 5 mM was added. Protein digestion was performed with a 1:100 Lys-C (Wako, 125-05061) to protein concentration for 5 h, followed by a 1:20 trypsin (V511A, Promega) to protein concentration for overnight digestion (16 h). Digestion with Lys-C and trypsin was carried out under gentle agitation at 37 °C. TMT labelling of peptides was performed according to the manufacturer’s protocol (Thermo Scientific, TMT 6 plex Mass Tag Labelling Kits and Reagents). For TMT labelling, half a 0.8 mg tube of TMT label was used to label a 45 µl sample with 50 µg peptides. A test mix of TMT-labelled samples was prepared by pooling 2 µl of each labelled sample. This was analysed by mass spectrometry, and the total intensity of all TMT tags was calculated. This allowed us to mix a precise 1:1 ratio of all 6 tags for the final experiment. In the final pooled sample of 50 µg peptides, SDC was removed by precipitation, by adding a final concentration of 2.5% formic acid, incubating for 10 min at room temperature, then sample was centrifuging at 13,000 rpm for 15 min. The supernatant was transferred to a fresh protein low-bind tube and evaporated to dryness in a speedvac. Peptides were reconstituted in 0.5% TFA then concentrated and cleaned up using DPX C18 pipette tips (DPX Technologies, XTR tips 10 mg C18AQ 300 Å) followed by evaporation in a speedvac. High pH reverse-phase fractionation [63] was performed on an Ultimate 3000 offline HPLC. C18-purified peptides were reconstituted in 20 mM ammonium formate pH 10 and loaded onto an RP column (Waters Acquity UPLC® BEH C18 1.7 µm 2.1 ×100 mm column). The samples were fractionated using a linear gradient of 0–60% B (90% ACN, 20 mM ammonium formate, pH 10) at 150 µl/min for 30 min. Twenty fractions were collected and pooled into 10 fractions using the mixing strategy Fr1 + Fr11, Fr2 + Fr12, and so on. Fractions were frozen at −80 °C. Samples were then dried in a speedvac and reconstituted in 2 µl 2% ACN 0.1% formic acid.

Peptides were analysed using a Thermo Scientific Orbitrap Fusion Lumos mass spectrometer coupled to a Thermo Scientific EASY-nLC 1200 nanoLC. Peptides were loaded onto a PepMap C18 EASY-spray column (2 µm, 100 Å, 75 mm × 50 cm) (Thermo Scientific). Peptides were fractionated using a 4–72% acetonitrile gradient in 0.1% formic acid over 140 min at a flow rate of 300 nl/min; 4–6% in 5 min, 6–32% in 120 min, 32–72% in 5 min, and isocratic for 5 min. Eluted TMT peptides were analysed using an SPS-MS3 method. MS1 was acquired in the Orbitrap (120k resolution with a scan range of 400–1400 m/z, AGC target 4.0 ×10^5^). MS2 was collected in the Ion Trap after CID fragmentation. MS3 was collected in the Orbitrap (HCD, 7.5 K resolution with AGC target 1.5 ×10^5^, and max IT 150). Raw data was analysed using MaxQuant (version 2.4.9.0) [64] with the integrated Andromeda search engine. MS/MS data was searched against the Uniprot Human database. An FDR of 0.01 was needed to give a protein identification.

Perseus (version 2.0.11) was used for statistical analysis. TMT intensities were log2 transformed. Proteins with less than 3 valid values were filtered out. Missing values were imputed from normal distribution with a down shift of 1.8. Volcano plot was created using a t-test and FDR of 0.05 and S0 as 0.1.

### Statistical analyses

Except for the mass spectrometry-based quantitative proteomic study, all experiments were conducted independently at least three times, yielding similar results. The mass spectrometry-based quantitative proteomic study was based on three independent biological replicates. The use of statistical analyses and sample numbers is described in the figure legends and in the Source Data file. Student’s *t* test was used for comparisons between two independent sample groups when the underlying distribution was undoubtedly normal. Otherwise, Welch’s *t* test was used.

## Supplementary information

S1

S2

S3

S4

S5

S6

S7

S8

S9

Original Data

Dataset 1

Source Data file

## Data Availability

The materials described in the manuscript, including all relevant raw data, will be freely available to any researcher wishing to use them for non-commercial purposes, without breaching participant confidentiality.

## References

[1] Li Y, Zhao L, Li XF. Hypoxia and the Tumor Microenvironment. Technol Cancer Res Treat. 2021;20:15330338211036304. 34350796 10.1177/15330338211036304 PMC8358492

[2] DeBerardinis RJ, Cheng T. Q’s next: the diverse functions of glutamine in metabolism, cell biology and cancer. Oncogene. 2010;29:313–24. 19881548 10.1038/onc.2009.358 PMC2809806

[3] Gatenby RA, Gillies RJ. Why do cancers have high aerobic glycolysis? Nat Rev Cancer. 2004;4:891–9. 15516961 10.1038/nrc1478

[4] Corbet C, Feron O. Tumour acidosis: from the passenger to the driver’s seat. Nat Rev Cancer. 2017;17:577–93. 28912578 10.1038/nrc.2017.77

[5] Lee P, Chandel NS, Simon MC. Cellular adaptation to hypoxia through hypoxia inducible factors and beyond. Nat Rev Mol Cell Biol. 2020;21:268–83. 32144406 10.1038/s41580-020-0227-y PMC7222024

[6] Webb BA, Chimenti M, Jacobson MP, Barber DL. Dysregulated pH: a perfect storm for cancer progression. Nat Rev Cancer. 2011;11:671–7. 21833026 10.1038/nrc3110

[7] Jing X, Yang F, Shao C, Wei K, Xie M, Shen H, et al. Role of hypoxia in cancer therapy by regulating the tumor microenvironment. Mol Cancer. 2019;18:157. 31711497 10.1186/s12943-019-1089-9 PMC6844052

[8] Gillies RJ, Pilot C, Marunaka Y, Fais S. Targeting acidity in cancer and diabetes. Biochim Biophys Acta Rev Cancer. 2019;1871:273–80. 30708040 10.1016/j.bbcan.2019.01.003 PMC6525044

[9] Mullen AR, Wheaton WW, Jin ES, Chen PH, Sullivan LB, Cheng T, et al. Reductive carboxylation supports growth in tumour cells with defective mitochondria. Nature. 2011;481:385–8. 22101431 10.1038/nature10642 PMC3262117

[10] Fendt SM, Bell EL, Keibler MA, Olenchock BA, Mayers JR, Wasylenko TM, et al. Reductive glutamine metabolism is a function of the alpha-ketoglutarate to citrate ratio in cells. Nat Commun. 2013;4:2236. 23900562 10.1038/ncomms3236 PMC3934748

[11] Spinelli JB, Yoon H, Ringel AE, Jeanfavre S, Clish CB, Haigis MC. Metabolic recycling of ammonia via glutamate dehydrogenase supports breast cancer biomass. Science. 2017;358:941–6. 29025995 10.1126/science.aam9305 PMC5748897

[12] Eng CH, Yu K, Lucas J, White E, Abraham RT. Ammonia derived from glutaminolysis is a diffusible regulator of autophagy. Sci Signal. 2010;3:ra31. 20424262 10.1126/scisignal.2000911

[13] Bell HN, Huber AK, Singhal R, Korimerla N, Rebernick RJ, Kumar R, et al. Microenvironmental ammonia enhances T cell exhaustion in colorectal cancer. Cell Metab. 2023;35:134–49.e6. 36528023 10.1016/j.cmet.2022.11.013 PMC9841369

[14] Hassell T, Gleave S, Butler M. Growth inhibition in animal cell culture. The effect of lactate and ammonia. Appl Biochem Biotechnol. 1991;30:29–41. 1952924 10.1007/BF02922022

[15] Schneider M, Marison IW, von Stockar U. The importance of ammonia in mammalian cell culture. J Biotechnol. 1996;46:161–85. 8672289 10.1016/0168-1656(95)00196-4

[16] Atanassov CL, Muller CD, Sarhan S, Knodgen B, Rebel G, Seiler N. Effect of ammonia on endocytosis, cytokine production and lysosomal enzyme activity of a microglial cell line. Res Immunol. 1994;145:277–88. 7824805 10.1016/s0923-2494(94)80016-2

[17] Kiesel VA, Sheeley MP, Donkin SS, Wendt MK, Hursting SD, Teegarden D. Increased Ammonium Toxicity in Response to Exogenous Glutamine in Metastatic Breast Cancer Cells. Metabolites. 2022;12:469. 10.3390/metabo12050469 PMC9145280 35629973

[18] Weber RA, Yen FS, Nicholson SPV, Alwaseem H, Bayraktar EC, Alam M, et al. Maintaining Iron Homeostasis Is the Key Role of Lysosomal Acidity for Cell Proliferation. Mol Cell. 2020;77:645–55.e7. 31983508 10.1016/j.molcel.2020.01.003 PMC7176020

[19] Allen WJ, Collinson I. A molecular dual carriageway. Elife. 2020;9:e61148. 10.7554/eLife.61148 PMC7447420 32840481

[20] Seglen PO, Grinde B, Solheim AE. Inhibition of the lysosomal pathway of protein degradation in isolated rat hepatocytes by ammonia, methylamine, chloroquine and leupeptin. Eur J Biochem. 1979;95:215–25. 456353 10.1111/j.1432-1033.1979.tb12956.x

[21] Casey JR, Grinstein S, Orlowski J. Sensors and regulators of intracellular pH. Nat Rev Mol Cell Biol. 2010;11:50–61. 19997129 10.1038/nrm2820

[22] Klionsky DJ, Eskelinen EL, Deretic V. Autophagosomes, phagosomes, autolysosomes, phagolysosomes, autophagolysosomes… wait, I’m confused. Autophagy. 2014;10:549–51. 24657946 10.4161/auto.28448 PMC4091142

[23] Hasilik A, von Figura K, Conzelmann E, Nehrkorn H, Sandhoff K. Lysosomal enzyme precursors in human fibroblasts. Activation of cathepsin D precursor in vitro and activity of beta-hexosaminidase A precursor towards ganglioside GM2. Eur J Biochem. 1982;125:317–21. 6214395 10.1111/j.1432-1033.1982.tb06685.x

[24] Brix K, Dunkhorst A, Mayer K, Jordans S. Cysteine cathepsins: cellular roadmap to different functions. Biochimie. 2008;90:194–207. 17825974 10.1016/j.biochi.2007.07.024

[25] Li Z, Ji X, Wang W, Liu J, Liang X, Wu H, et al. Ammonia Induces Autophagy through Dopamine Receptor D3 and MTOR. PLoS One. 2016;11:e0153526. 27077655 10.1371/journal.pone.0153526 PMC4831814

[26] Harder LM, Bunkenborg J, Andersen JS. Inducing autophagy: a comparative phosphoproteomic study of the cellular response to ammonia and rapamycin. Autophagy. 2014;10:339–55. 24300666 10.4161/auto.26863 PMC5396081

[27] Cheong H, Lindsten T, Wu J, Lu C, Thompson CB. Ammonia-induced autophagy is independent of ULK1/ULK2 kinases. Proc Natl Acad Sci USA. 2011;108:11121–6. 21690395 10.1073/pnas.1107969108 PMC3131371

[28] Mi H, Muruganujan A, Huang X, Ebert D, Mills C, Guo X, et al. Protocol Update for large-scale genome and gene function analysis with the PANTHER classification system (v.14.0). Nat Protoc. 2019;14:703–21. 30804569 10.1038/s41596-019-0128-8 PMC6519457

[29] Klionsky DJ, Abdel-Aziz AK, Abdelfatah S, Abdellatif M, Abdoli A, Abel S, et al. Guidelines for the use and interpretation of assays for monitoring autophagy (4th edition)(1). Autophagy. 2021;17:1–382. 33634751 10.1080/15548627.2020.1797280 PMC7996087

[30] Zhen Y, Stenmark H. Autophagosome Biogenesis. Cells. 2023;12:668. 10.3390/cells12040668 PMC9954227 36831335

[31] Johansen T, Lamark T. Selective Autophagy: ATG8 Family Proteins, LIR Motifs and Cargo Receptors. J Mol Biol. 2020;432:80–103. 31310766 10.1016/j.jmb.2019.07.016

[32] Mejlvang J, Olsvik H, Svenning S, Bruun JA, Abudu YP, Larsen KB, et al. Starvation induces rapid degradation of selective autophagy receptors by endosomal microautophagy. J Cell Biol. 2018;217:3640–55. 30018090 10.1083/jcb.201711002 PMC6168274

[33] Durgan J, Florey O. Many roads lead to CASM: Diverse stimuli of noncanonical autophagy share a unifying molecular mechanism. Sci Adv. 2022;8:eabo1274. 36288315 10.1126/sciadv.abo1274 PMC9604613

[34] Dooley HC, Razi M, Polson HE, Girardin SE, Wilson MI, Tooze SA. WIPI2 links LC3 conjugation with PI3P, autophagosome formation, and pathogen clearance by recruiting Atg12-5-16L1. Mol Cell. 2014;55:238–52. 24954904 10.1016/j.molcel.2014.05.021 PMC4104028

[35] Robbins E, Marcus PI. Dynamics of Acridine Orange-Cell Interaction. I. Interrelationships of Acridine Orange Particles and Cytoplasmic Reddening. J Cell Biol. 1963;18:237–50. 14079487 10.1083/jcb.18.2.237 PMC2106306

[36] Corbet C, Draoui N, Polet F, Pinto A, Drozak X, Riant O, et al. The SIRT1/HIF2alpha axis drives reductive glutamine metabolism under chronic acidosis and alters tumor response to therapy. Cancer Res. 2014;74:5507–19. 25085245 10.1158/0008-5472.CAN-14-0705

[37] Levin-Konigsberg R, Mitra K, Nigam A, Spees K, Hivare P, Liu K, et al. SLC12A9 is a lysosome-detoxifying ammonium - chloride co-transporter. bioRxiv. 2023, https://www.biorxiv.org/content/10.1101/2023.05.22.541801v1.

[38] Yang C, Wang X. Lysosome biogenesis: Regulation and functions. J Cell Biol. 2021;220:e202102001. 10.1083/jcb.202102001 PMC8105738 33950241

[39] Ellegaard AM, Bach P, Jaattela M. Targeting Cancer Lysosomes with Good Old Cationic Amphiphilic Drugs. Rev Physiol Biochem Pharm. 2023;185:107–52. 10.1007/112_2020_56 33398504

[40] Seglen PO. Effects of amino acids, ammonia and leupeptin on protein synthesis and degradation in isolated rat hepatocytes. Biochem J. 1978;174:469–74. 708399 10.1042/bj1740469 PMC1185936

[41] Hwang JR, Kim WY, Cho YJ, Ryu JY, Choi JJ, Jeong SY, et al. Chloroquine reverses chemoresistance via upregulation of p21(WAF1/CIP1) and autophagy inhibition in ovarian cancer. Cell Death Dis. 2020;11:1034. 33277461 10.1038/s41419-020-03242-x PMC7718923

[42] Pellegrini P, Strambi A, Zipoli C, Hagg-Olofsson M, Buoncervello M, Linder S, et al. Acidic extracellular pH neutralizes the autophagy-inhibiting activity of chloroquine: implications for cancer therapies. Autophagy. 2014;10:562–71. 24492472 10.4161/auto.27901 PMC3984580

[43] Mauthe M, Orhon I, Rocchi C, Zhou X, Luhr M, Hijlkema KJ, et al. Chloroquine inhibits autophagic flux by decreasing autophagosome-lysosome fusion. Autophagy. 2018;14:1435–55. 29940786 10.1080/15548627.2018.1474314 PMC6103682

[44] Webb BA, Aloisio FM, Charafeddine RA, Cook J, Wittmann T, Barber DL. pHLARE: a new biosensor reveals decreased lysosome pH in cancer cells. Mol Biol Cell. 2021;32:131–42. 33237838 10.1091/mbc.E20-06-0383 PMC8120692

[45] Byrne MB, Leslie MT, Gaskins HR, Kenis PJA. Methods to study the tumor microenvironment under controlled oxygen conditions. Trends Biotechnol. 2014;32:556–63. 25282035 10.1016/j.tibtech.2014.09.006 PMC4254115

[46] Helmlinger G, Sckell A, Dellian M, Forbes NS, Jain RK. Acid production in glycolysis-impaired tumors provides new insights into tumor metabolism. Clin Cancer Res. 2002;8:1284–91. 11948144

[47] Tang X, Lucas JE, Chen JL, LaMonte G, Wu J, Wang MC, et al. Functional interaction between responses to lactic acidosis and hypoxia regulates genomic transcriptional outputs. Cancer Res. 2012;72:491–502. 22135092 10.1158/0008-5472.CAN-11-2076 PMC3261313

[48] Sorensen BS, Alsner J, Overgaard J, Horsman MR. Hypoxia induced expression of endogenous markers in vitro is highly influenced by pH. Radiother Oncol. 2007;83:362–6. 17512623 10.1016/j.radonc.2007.04.028

[49] Jin J, Byun JK, Choi YK, Park KG. Targeting glutamine metabolism as a therapeutic strategy for cancer. Exp Mol Med. 2023;55:706–15. 37009798 10.1038/s12276-023-00971-9 PMC10167356

[50] Wojtkowiak JW, Rothberg JM, Kumar V, Schramm KJ, Haller E, Proemsey JB, et al. Chronic autophagy is a cellular adaptation to tumor acidic pH microenvironments. Cancer Res. 2012;72:3938–47. 22719070 10.1158/0008-5472.CAN-11-3881 PMC3749826

[51] Tan Q, Wang M, Yu M, Zhang J, Bristow RG, Hill RP, et al. Role of Autophagy as a Survival Mechanism for Hypoxic Cells in Tumors. Neoplasia. 2016;18:347–55. 27292024 10.1016/j.neo.2016.04.003 PMC4909700

[52] Trakarnphornsombat W, Kimura H. Live-cell tracking of gamma-H2AX kinetics reveals the distinct modes of ATM and DNA-PK in the immediate response to DNA damage. J Cell Sci. 2023;136:jcs260698. 10.1242/jcs.260698 PMC10163350 36999484

[53] Mladenov E, Fan X, Dueva R, Soni A, Iliakis G. Radiation-dose-dependent functional synergisms between ATM, ATR and DNA-PKcs in checkpoint control and resection in G(2)-phase. Sci Rep. 2019;9:8255. 31164689 10.1038/s41598-019-44771-6 PMC6547644

[54] Cho YY, Kwon OH, Chung S. Preferred Endocytosis of Amyloid Precursor Protein from Cholesterol-Enriched Lipid Raft Microdomains. Molecules. 2020;25:5490. 10.3390/molecules25235490 PMC7727664 33255194

[55] Komatsu A, Iida I, Nasu Y, Ito G, Harada F, Kishikawa S, et al. Ammonia induces amyloidogenesis in astrocytes by promoting amyloid precursor protein translocation into the endoplasmic reticulum. J Biol Chem. 2022;298:101933. 35427648 10.1016/j.jbc.2022.101933 PMC9117890

[56] Ni HM, Bockus A, Wozniak AL, Jones K, Weinman S, Yin XM, et al. Dissecting the dynamic turnover of GFP-LC3 in the autolysosome. Autophagy. 2011;7:188–204. 21107021 10.4161/auto.7.2.14181 PMC3039769

[57] Xu H, Ren D. Lysosomal physiology. Annu Rev Physiol. 2015;77:57–80. 25668017 10.1146/annurev-physiol-021014-071649 PMC4524569

[58] Hu M, Li P, Wang C, Feng X, Geng Q, Chen W, et al. Parkinson’s disease-risk protein TMEM175 is a proton-activated proton channel in lysosomes. Cell. 2022;185:2292–308.e20. 35750034 10.1016/j.cell.2022.05.021 PMC9236176

[59] Ratto E, Chowdhury SR, Siefert NS, Schneider M, Wittmann M, Helm D, et al. Direct control of lysosomal catabolic activity by mTORC1 through regulation of V-ATPase assembly. Nat Commun. 2022;13:4848. 35977928 10.1038/s41467-022-32515-6 PMC9385660

[60] Collins MP, Stransky LA, Forgac M. AKT Ser/Thr kinase increases V-ATPase-dependent lysosomal acidification in response to amino acid starvation in mammalian cells. J Biol Chem. 2020;295:9433–44. 32409581 10.1074/jbc.RA120.013223 PMC7363138

[61] Zhang J, Zeng W, Han Y, Lee WR, Liou J, Jiang Y. Lysosomal LAMP proteins regulate lysosomal pH by direct inhibition of the TMEM175 channel. Mol Cell. 2023;83:2524–39.e7. 37390818 10.1016/j.molcel.2023.06.004 PMC10528928

[62] Xiong J, Luu TTT, Venkatachalam K, Du G, Zhu MX. Glutamine Produces Ammonium to Tune Lysosomal pH and Regulate Lysosomal Function. Cells. 2022;12:80. 10.3390/cells12010080 PMC9819001 36611873

[63] Stein DR, Hu X, McCorrister SJ, Westmacott GR, Plummer FA, Ball TB, et al. High pH reversed-phase chromatography as a superior fractionation scheme compared to off-gel isoelectric focusing for complex proteome analysis. Proteomics. 2013;13:2956–66. 23956148 10.1002/pmic.201300079

[64] Tyanova S, Temu T, Cox J. The MaxQuant computational platform for mass spectrometry-based shotgun proteomics. Nat Protoc. 2016;11:2301–19. 27809316 10.1038/nprot.2016.136

