# Supplementary material for: Low extracellular pH protects cancer cells from ammonia toxicity
Source: Cell Death Discov. 2025 Apr 3;11:137. doi: 10.1038/s41420-025-02440-w (PMC11968834; doi:10.1038/s41420-025-02440-w)

Original western blots for figure 2D

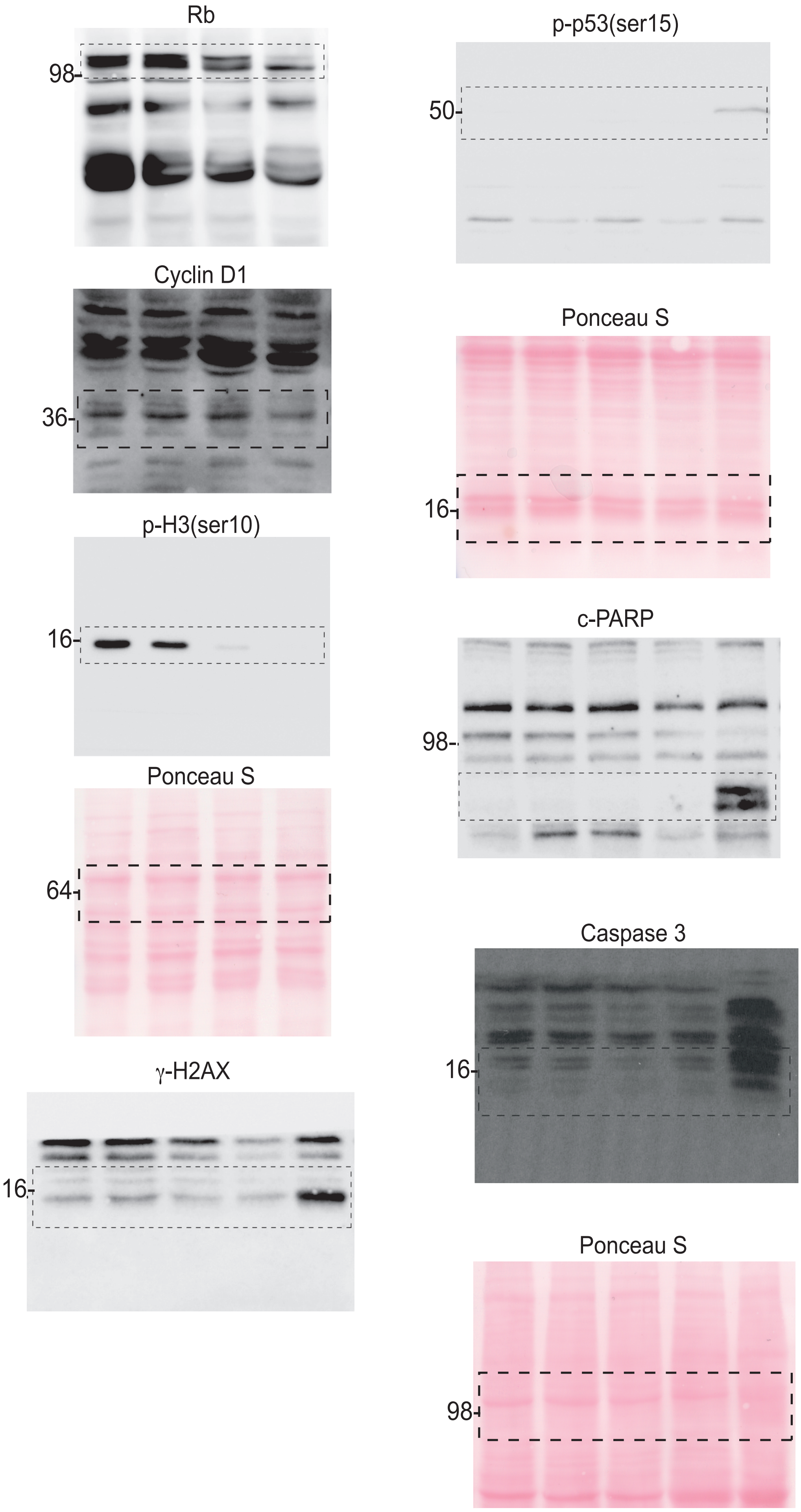

Original western blots for figure 4a

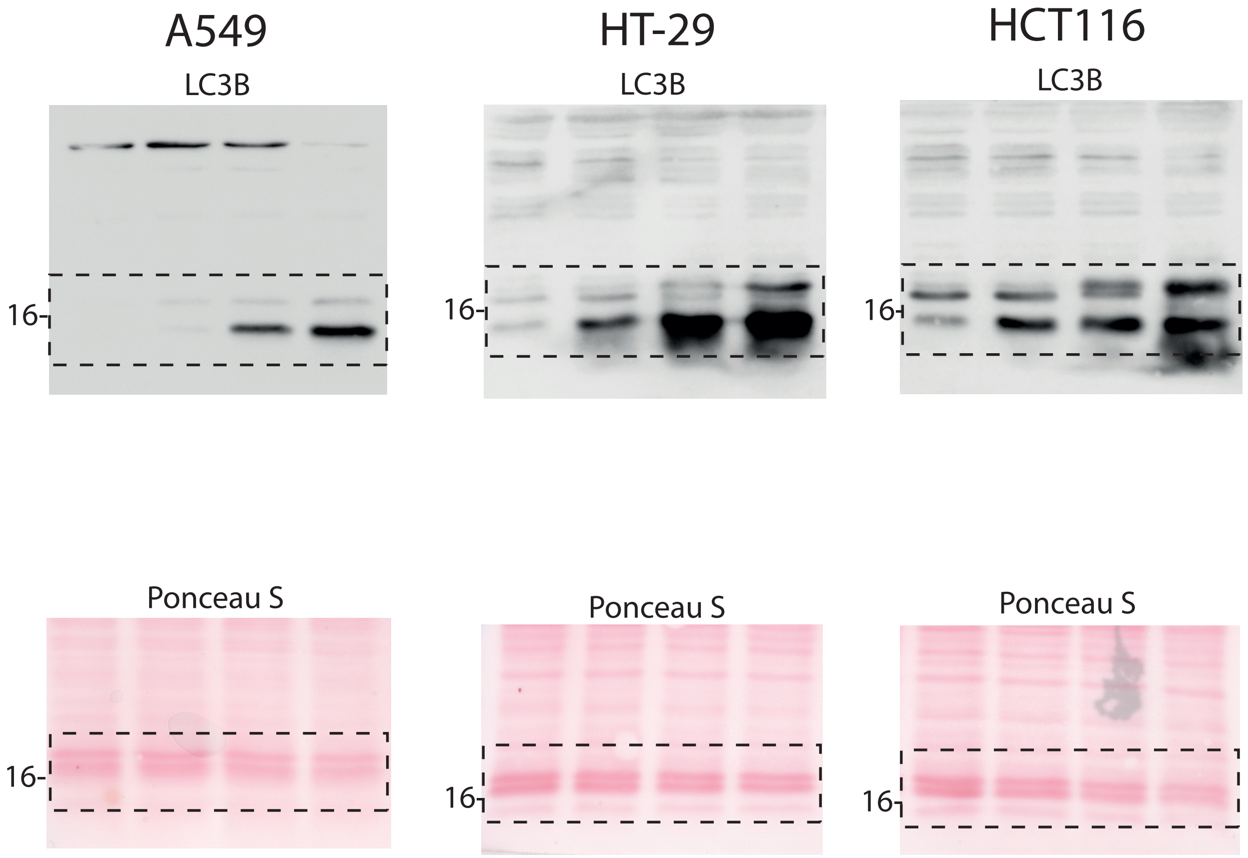

Original western blots for figure 5a

NBR1

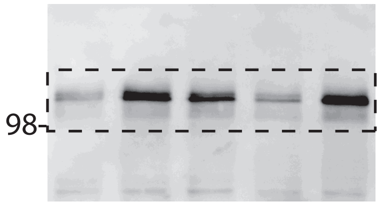

p62

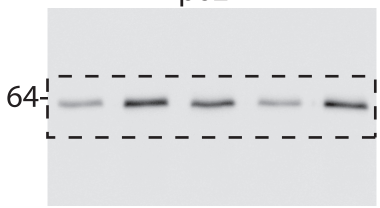

LC3B

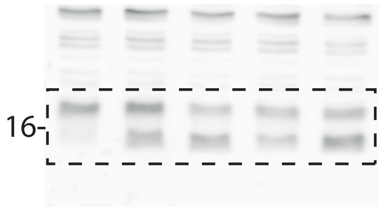

Ponceau S

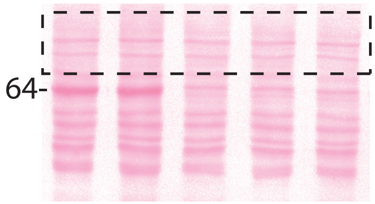

Original western blots for figure 5b

NBR1

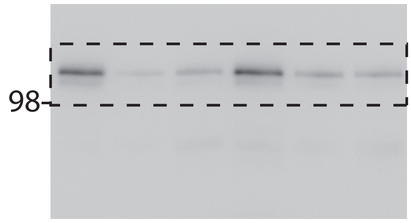

p62

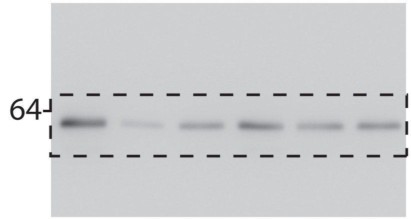

NCOA4

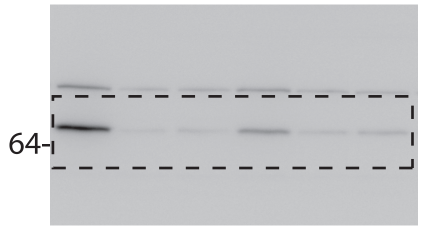

LC3B

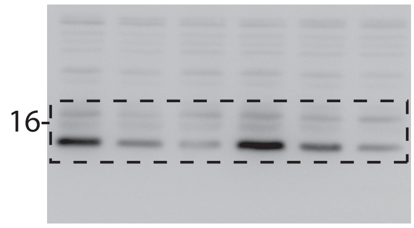

Ponceau S

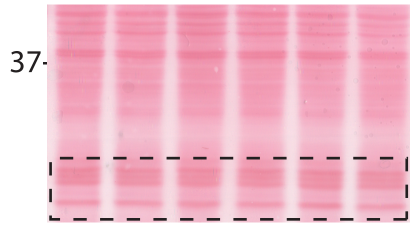

Original western blots for figure 5c

NBR1

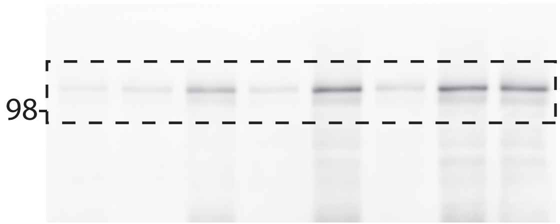

LC3B

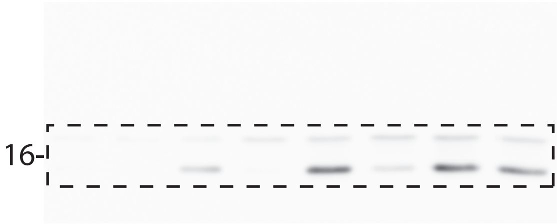

p62

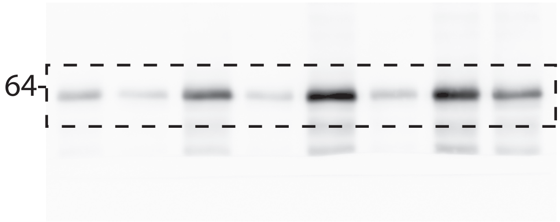

Cathepsin D

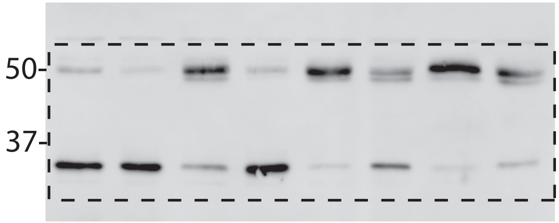

NCOA4

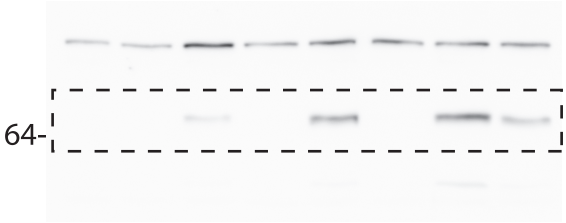

Ponceau S

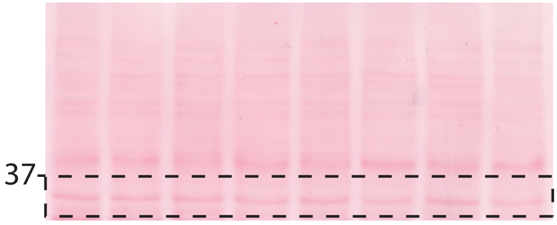

Original western blots for figure s2 (HCT116)

Rb

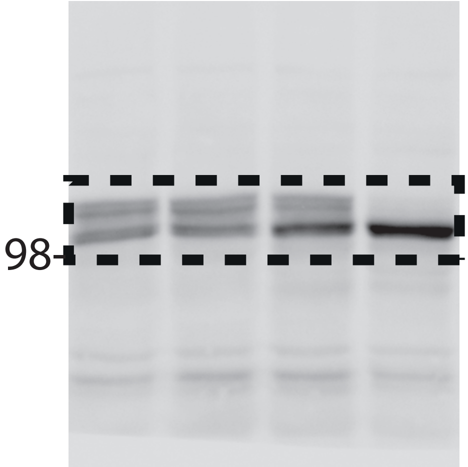

p-H3(ser10)

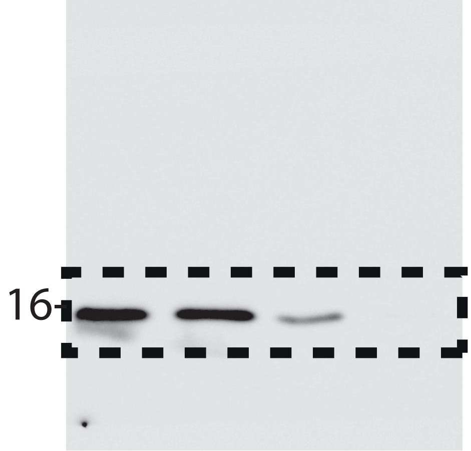

p-p53(ser15)

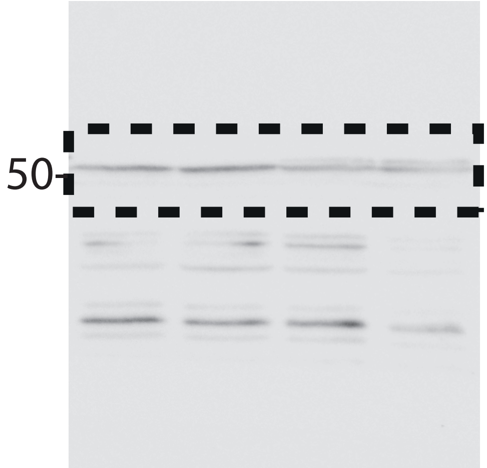

γH2AX

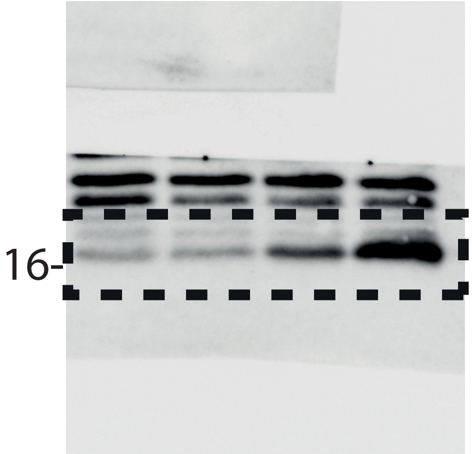

c-PARP

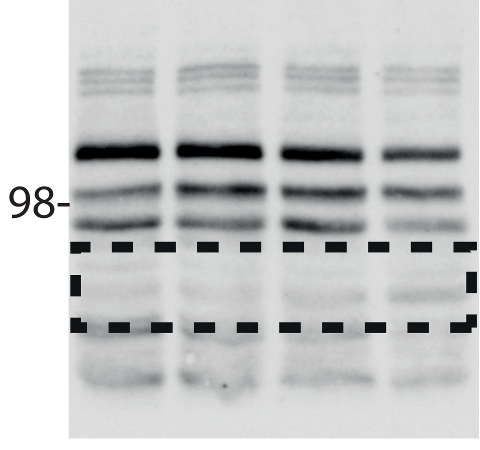

Ponceau S

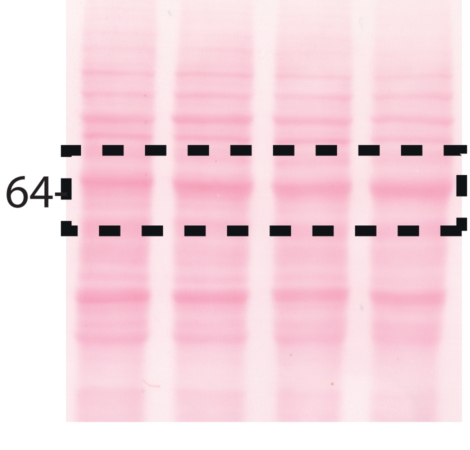

Original western blots for figure s2 (HT29)

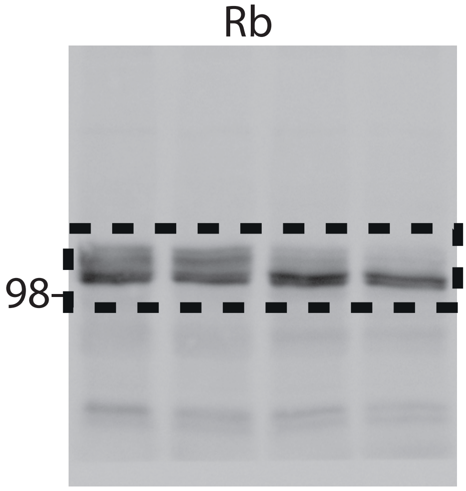

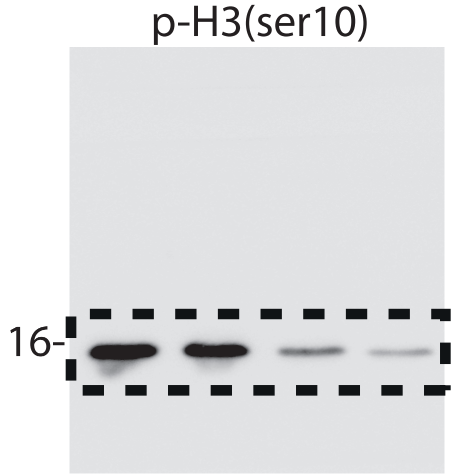

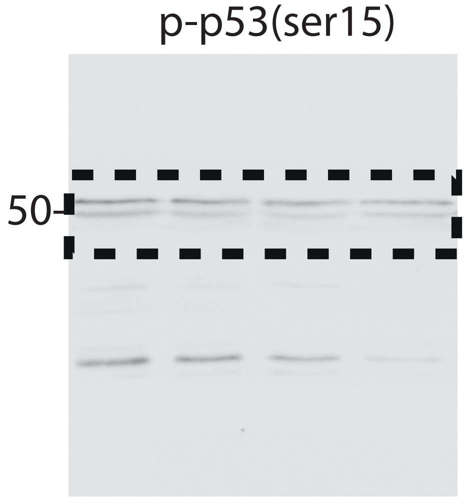

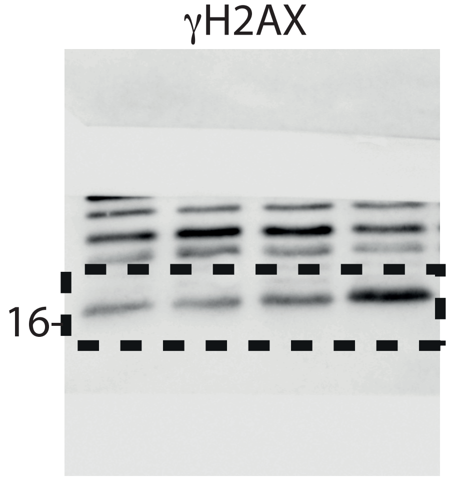

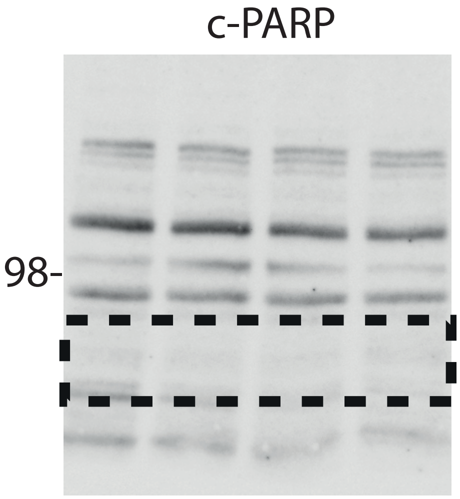

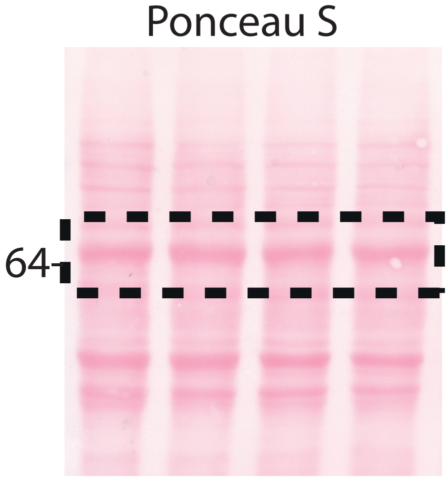

Original western blots for figure s4

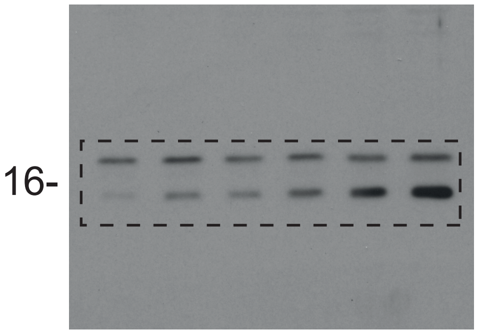

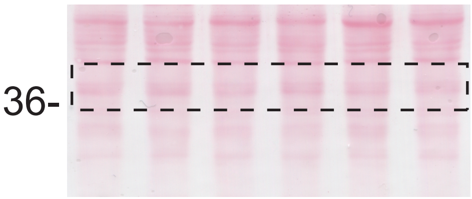

Supplement: Supplementary file 10 — Original Data [file 41420_2025_2440_MOESM10_ESM.pdf]
